# miR302a and 122 are deregulated in small extracellular vesicles from ARPE-19 cells cultured with H_2_O_2_

**DOI:** 10.1038/s41598-019-54373-x

**Published:** 2019-11-29

**Authors:** Maria Oltra, Lorena Vidal-Gil, Rosa Maisto, Sara S. Oltra, Francisco Javier Romero, Javier Sancho-Pelluz, Jorge Miguel Barcia

**Affiliations:** 1 Escuela de Doctorado Universidad Católica de Valencia San Vicente Mártir, Valencia, Spain; 2 Neurobiología y Neurofisiología, Facultad de Medicina y Ciencias de la Salud, Universidad Católica de Valencia San Vicente Mártir, Valencia, Spain; 3 Centro de Investigación Traslacional San Alberto Magno, Universidad Católica de Valencia San Vicente Mártir, Valencia, Spain; 4 Università degli studi della Campania Luigi Vanvitelli, Naples, Italy; 5 INCLIVA Biomedical Research Institute, Hospital Clínico Universitario Valencia, University of Valencia, Valencia, Spain; 6 Hospital General de Requena, Valencia, Spain

**Keywords:** Multivesicular bodies, Macular degeneration

## Abstract

Age related macular degeneration (AMD) is a common retina-related disease leading to blindness. Little is known on the origin of the disease, but it is well documented that oxidative stress generated in the retinal pigment epithelium and choroid neovascularization are closely involved. The study of circulating miRNAs is opening new possibilities in terms of diagnosis and therapeutics. miRNAs can travel associated to lipoproteins or inside small Extracellular Vesicles (sEVs). A number of reports indicate a significant deregulation of circulating miRNAs in AMD and experimental approaches, but it is unclear whether sEVs present a significant miRNA cargo. The present work studies miRNA expression changes in sEVs released from ARPE-19 cells under oxidative conditions (i.e. hydrogen peroxide, H_2_O_2_). H_2_O_2_ increased sEVs release from ARPE-19 cells. Moreover, 218 miRNAs could be detected in control and H_2_O_2_ induced-sEVs. Interestingly, only two of them (hsa-miR-302a and hsa-miR-122) were significantly under-expressed in H_2_O_2_-induced sEVs. Results herein suggest that the down regulation of miRNAs 302a and 122 might be related with previous studies showing sEVs-induced neovascularization after oxidative challenge in ARPE-19 cells.

## Introduction

microRNA (miRNA) are small non-coding RNA sequences (21-25 nucleotides) able to regulate the expression of one or more mRNAs^1,2^. miRNAs regulate protein translation by targeting their complementary mRNAs and by repressing translation or degrading a target mRNA. Nowadays, miRNA research has been rocketed from 214 publications in 2014 to 11,610 in 2018 (source: Pubmed). Many clinical fields are now focused on the study of miRNA for diagnosis or treatment purposes^3,4^. Moreover, miRNAs play an important role in different cellular processes such as angiogenesis^5,6^, oxidative stress^7^, and immune responses^8^.

Most of the cells can release extracellular vesicles (EVs), which may interact with both neighbouring or distant target cells^9,10^. EVs typically include genetic material and proteins, making these vesicles key in cell to cell communication^11^. Among these EVs, are the small EVs (sEVs; <100 nm diameter)^12^, which were observed to carry miRNAs^13^. Cargo of sEVs depends on the cellular origin and the homeostatic state^14^.

Recent studies analysed miRNA expression in biological samples from patients with age related macular degeneration (AMD), in order to identify those miRNAs related to the pathophysiology and progression of the disease. Among these, miR-9, miR-23a, miR-27a, miR-34a, miR-146a, miR-155 have been proposed as potential candidates^15–19^. The “wet” form of AMD is characterized by neovascularization^20^. The retinal pigment epithelium (RPE) plays a pivotal role between the photoreceptor cell layer and the choroid, the vascular network surrounding the eye. This interaction is critical for retinal homeostasis^21^. Ren and collaborators analysed circulating miRNAs from AMD patients and proposed miR-27a-3p, miR-29b-3p, and miR-195-5p as candidate biomarkers for AMD diagnosis^19^.

One of the most challenging issues in ophthalmological sciences is to identify early markers for AMD in order to prevent its clinical evolution. To date AMD treatments are limited to diminish the evolution of the disease as anti-VEGF drugs (eg. Bevacizumab)^22^. The present work studies the oxidative-induced response in RPE cells, in order to find potential miRNAs related to neovascularization within sEVs.

## Results

### H_2_O_2_-induced oxidative stress in ARPE-19 cells

**Figure 1:**
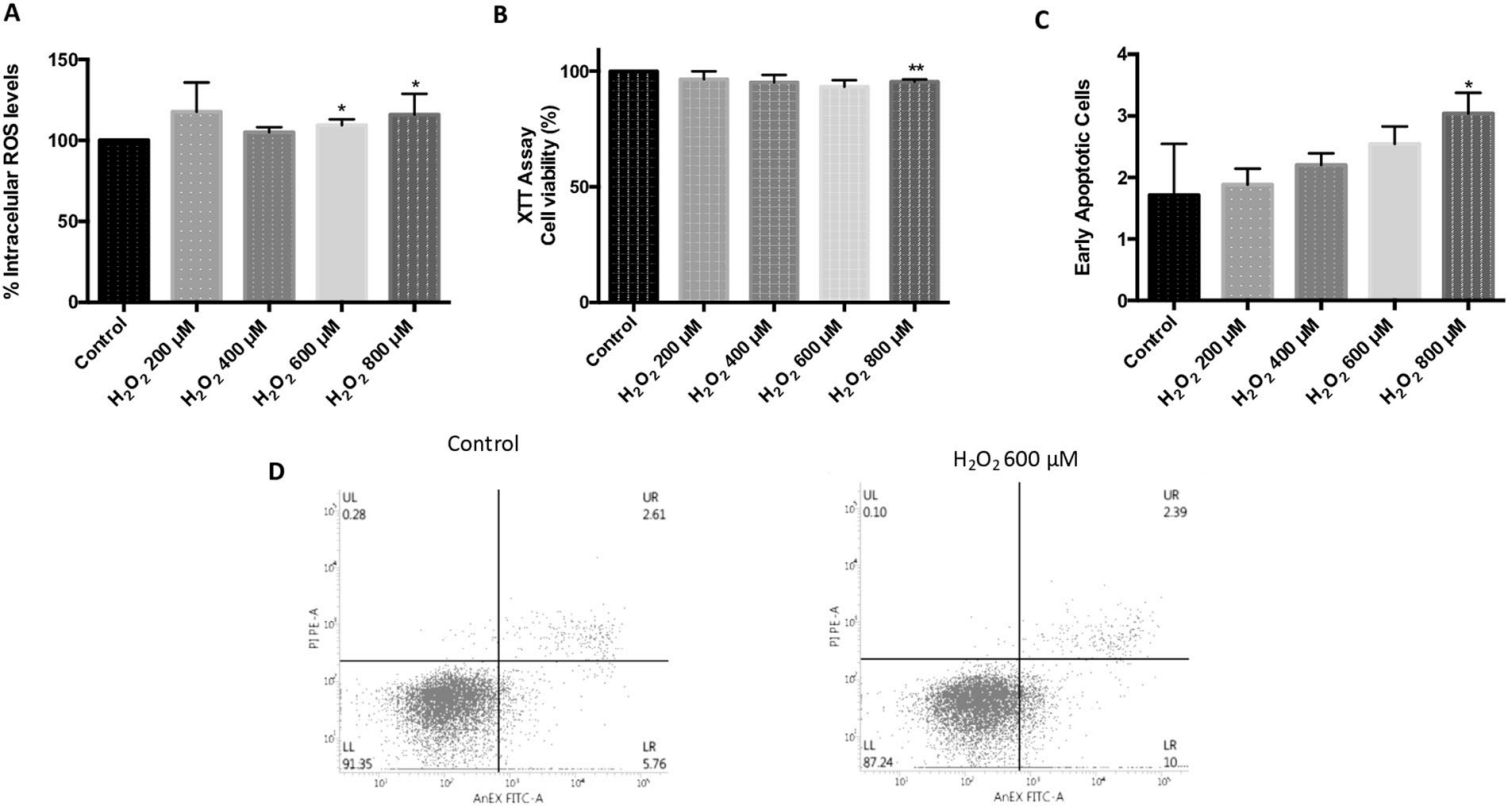
Effect of oxidative stress in ARPE-19 cells. Superoxide anions were measured by DHE after 24 h of H_2_O_2_ treatment. (**A**) XTT cell viability as percentage to control. (**B**) Early apoptotic cells measured using AnnexinV-IP. (**C**) Flow cytometry histograms: alive cells (annexin V^−^, PI^−^), early apoptosis (annexin V^+^, PI^−^) and necrotic cells (annexin V^+^, IP^+^). (**D**) Values are expressed as mean ± SEM (n = 3). Statistically significant differences were set at *p < 0.05 and **p < 0.01.

ARPE-19 cells exposed for 24 h to H_2_O_2_ significantly increased intracellular reactive oxygen species (ROS) levels when using a concentration of 600 μM (106 ± 1.69) and 800 μM (103.1 ± 0.9892), compared to control (100 ± 0). Nevertheless, 400 μM H_2_O_2_, or lower, did not increase ROS levels compared to control (Fig. 1A). In order to assess whether H_2_O_2_-induced ROS affected ARPE-19 cell viability, a XTT assay was performed. Cell viability was significantly decreased when using 800 μM H_2_O_2_ (95.45 ± 0.9999) but not with lower concentrations (Fig. 1B). Moreover, 800 μM H_2_O_2_ concentration also increased early apoptosis (3.038 ± 0.1689) with respect to control (1.713 ± 0.4796) (Fig. 1C,D).

### H_2_O_2_ increased sEVs release in ARPE-19

**Figure 2:**
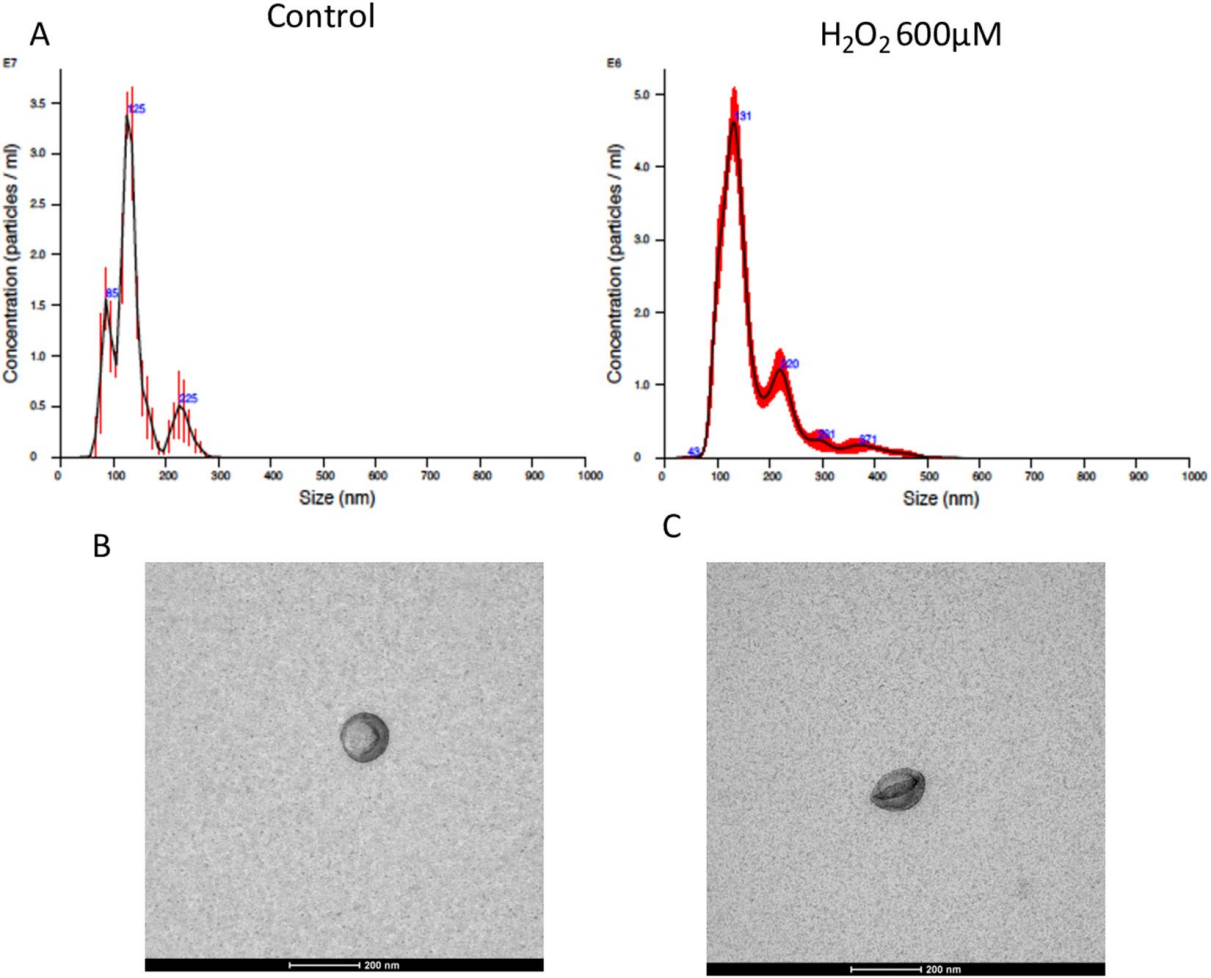
Characterization of sEVs released by ARPE-19 cells. Size-distribution analysis and sEVs number were performed by Nanoparticle Tracking Analysis. (**A**) EVs released by ARPE-19 cells control (**B**) and ARPE-19 cells treated by 600 μM H_2_O_2_ (**C**) were detected by electron microscopy.

Matching previous results on cell viability (see Fig. 1), 600 µM H_2_O_2_ was used to stress ARPE-19 cells without killing them (sub-lethal concentration). After 24 h of 600 µM H_2_O_2_ exposure, ARPE-19 cells significantly increased the number of EVs released to the medium (Fig. 2). More precisely, a 40% increase in the release of EVs was observed after 600 µM H_2_O_2_ exposure, compared to controls. Number and size of EVs were studied using a nanoparticle tracking system (NanoSight) (Fig. 2A). Besides, EVs were observed using transmission electron microscopy (TEM), which showed that, according to size and morphology, most of the EVs observed can be classified as sEVs (Fig. 2B,C).

### miRNA Expression in ARPE-19 cells and sEVs

**Figure 3:**
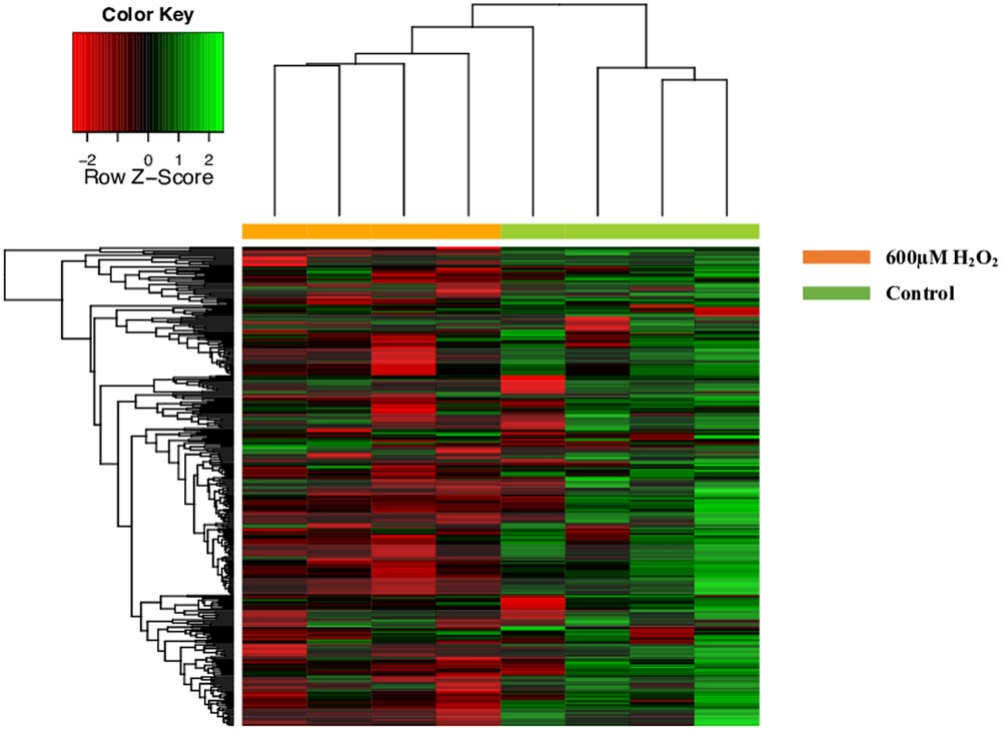
Heatmap of miRNA expression profile from array assay of ARPE-19 cells. After 24 h of 600 μM H_2_O_2,_ the miRNA expression profile was analyzed, using an array for microRNA. Hierarchical unsupervised clustering was performed with the expressed miRNAs in ARPE-19 cells. Each column represents an individual cell sample. Orange columns are ARPE-19 cells treat with 600 μM H_2_O_2_ and green columns ARPE-19 cell controls. Control sample (n = 4) and 600 μM H_2_O_2_ sample (n = 4). The overexpressed miRNAs are shown in green and under-expressed miRNAs in red. Expression values are calculated with respect to the reference genes of the array assay.

**Figure 4:**
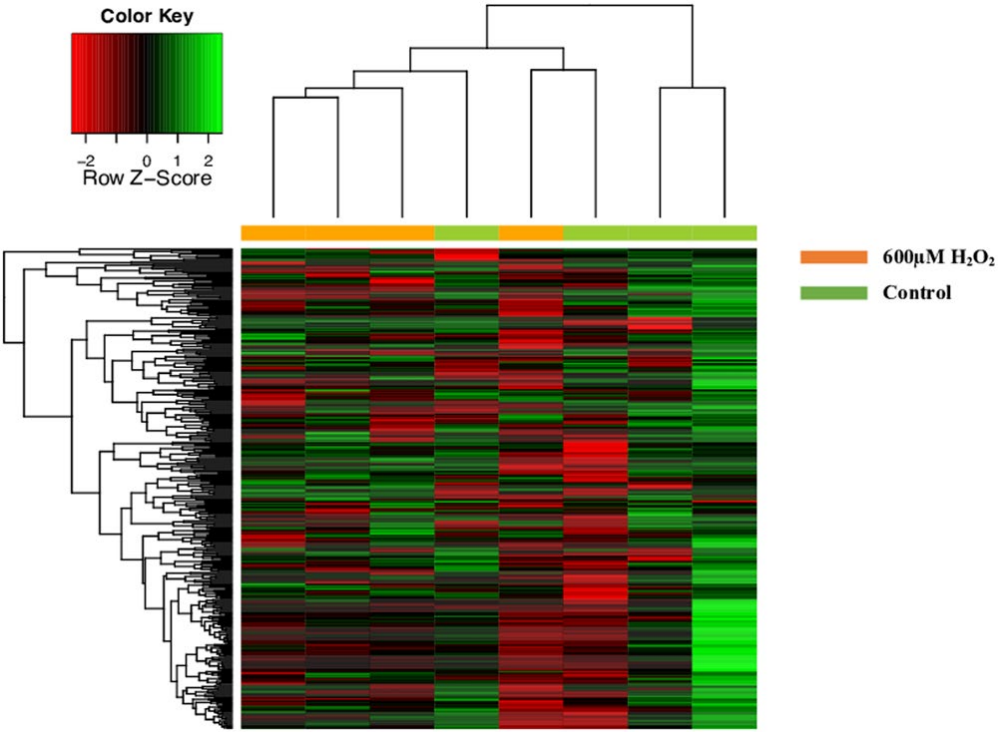
Heatmap of miRNA expression profile from array assay of the sEVs released by ARPE-19 cells. After 24 h of 600 μM H_2_O_2_, the miRNA expression profile of sEVs was analyzed, using an array of microRNA. Hierarchical unsupervised clustering was performed with the expressed miRNAs in ARPE-19-released sEVs. Each column represents an individual cell sample. Orange columns are sEVs released by ARPE-19 cells treat with 600 μM H_2_O_2_ and green columns are sEVs released by ARPE-19 control cells. Control sample (n = 4) and 600 μM H_2_O_2_ sample (n = 4). Overexpressed miRNAs (green) and under-expressed miRNAs (red). The expression values are calculated with respect to the reference genes of the array.

In the present study, 384 miRNAs were analysed, and two different miRNA clusters could be set after hierarchical clustering (Fig. 3). The same proceeding was performed with the extracellular medium to collect sEVs and miRNAs providing also two clusters (Fig. 4).

#### miRNA Expression in ARPE-19 cells (Cell miRNA)

**Figure 5:**
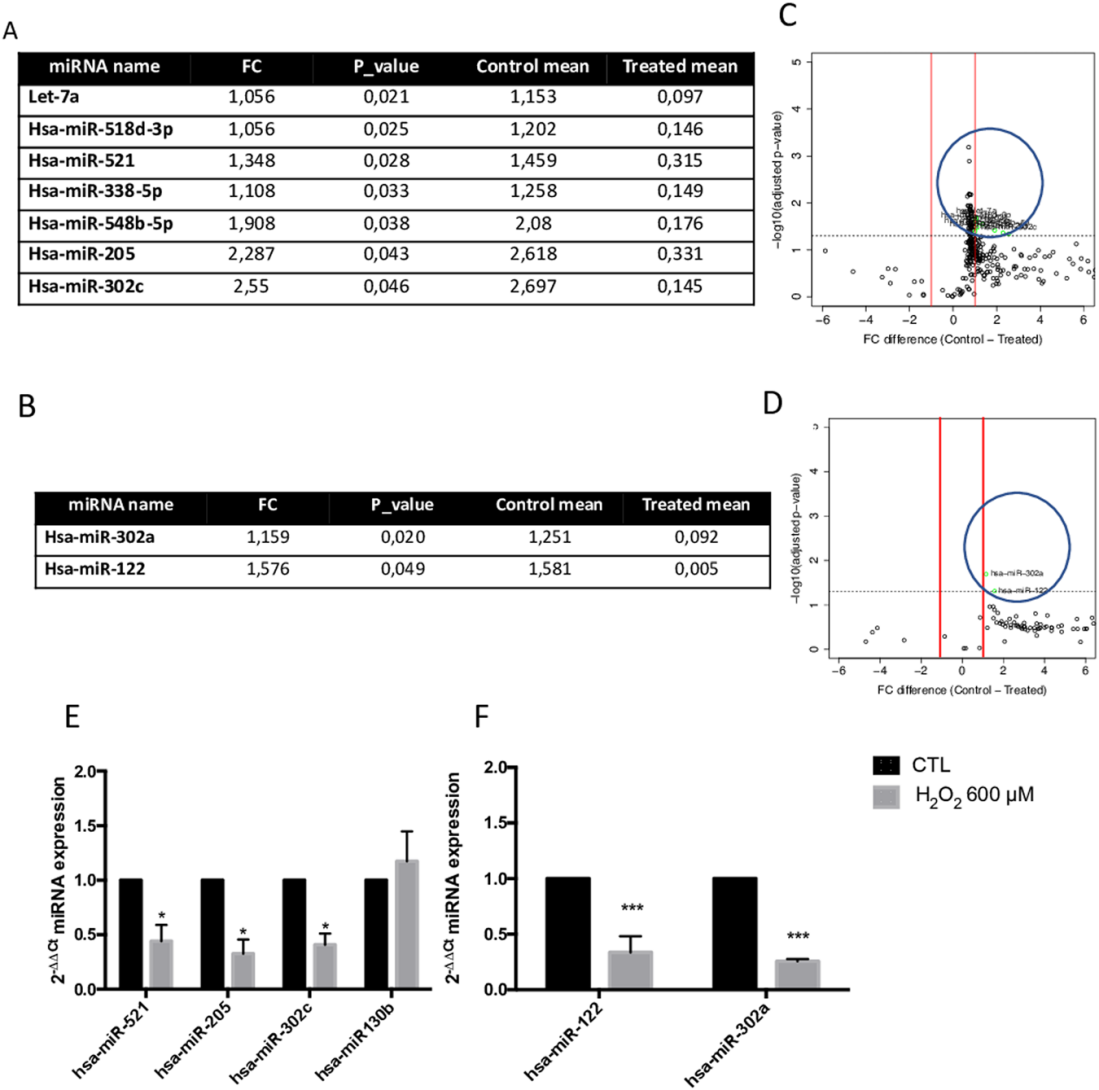
miRNAs significantly regulated in ARPE-19 cells. Cell miRNAs (**A**) and sEV miRNA (**B**) up-regulated in ARPE-19 cells with fold change (FC) > 1. Volcano plot of miRNA expression profile. The X axis represent the miRNA expression level and the Y axis represents p-value (T-student Test). The red lines indicate FC ± 1 P < 0.05 (**C**,**D**). Relative expression of the selected Cell miRNAs (**E**) and sEV miRNAs (**F**) with FC > 1 (control vs 600 μM H_2_O_2_). miRNA expression was quantified by qRT-PCR and calculate using the 2^−ΔΔCt^. The p-value was calculated by Two-way ANOVA. Results are expressed as mean ± SEM of n = 3–4. *p-value < 0.05 vs Control and ***p-value < 0.001.

As shown in Fig. 3, there is a significant miRNA repression after H_2_O_2_ treatment when compared with control ARPE-19 cells. As a result, 306 out of 384 Cell miRNAs were detected by the array. From those, 59 Cell miRNAs were significantly expressed in ARPE-19 cells (*p* < 0.05) (Table 1). Moreover, seven out of 59 Cell miRNAs were significantly under-expressed in H_2_O_2_-treated cells (fold change, FC > 1) than in control conditions. Different detected miRNAs are shown in Fig. 5A,C. It is noteworthy to underline that hsa-miR-205 and hsa-miR-302c presented a dramatic decrease in treated cells when compared to others (FC > 2).

**Table 1:** miRNAs significantly regulated in ARPE-19 cells under oxidative stress. The miRNAs are ranged according to the P-value.

miRNA	FC	P_value	miRNA	FC	P_value
**hsa-miR-139-3p**	0,71	0,00065	**hsa-miR-371-5p**	0,95	0,02665
**hsa-miR-192**	0,74	0,00129	**hsa-miR-92a**	0,70	0,02672
**hsa-miR-22**	0,73	0,00646	**hsa-miR-361-5p**	0,79	0,02674
**hsa-miR-15b**	0,73	0,00657	**hsa-miR-521**	1,35	0,02850
**hsa-let-7c**	0,82	0,00661	**hsa-miR-30c**	0,78	0,02935
**hsa-let-7b**	0,77	0,00670	**hsa-miR-335**	0,85	0,03051
**hsa-miR-221**	0,66	0,00723	**hsa-miR-28-5p**	0,94	0,03170
**hsa-miR-323-5p**	0,79	0,01131	**hsa-let-7g**	0,84	0,03204
**hsa-miR-15a**	0,67	0,01139	**hsa-miR-29c**	0,82	0,03218
**hsa-miR-18b**	0,70	0,01164	**hsa-miR-488**	0,98	0,03271
**hsa-miR-151-3p**	0,78	0,01193	**hsa-miR-532-3p**	0,86	0,03316
**hsa-miR-183**	0,80	0,01214	**hsa-miR-338-5p**	1,11	0,03332
**hsa-miR-518b**	0,76	0,01364	**hsa-miR-125a-5p**	0,82	0,03352
**hsa-miR-10a**	0,78	0,01407	**hsa-miR-499-3p**	0,72	0,03376
**hsa-miR-23a**	0,79	0,01456	**hsa-miR-29a**	0,72	0,03509
**hsa-miR-151-5p**	0,85	0,01492	**hsa-miR-20b**	0,81	0,03536
**hsa-miR-224**	0,78	0,01537	**hsa-miR-148a**	0,90	0,03727
**hsa-miR-186**	0,52	0,01561	**hsa-miR-548b-5p**	1,91	0,03849
**hsa-let-7f**	0,86	0,01619	**hsa-miR-218**	1,00	0,04031
**hsa-miR-27a**	0,80	0,01739	**hsa-miR-505**	0,65	0,04090
**hsa-miR-148b**	0,66	0,01813	**hsa-miR-205**	2,29	0,04350
**hsa-miR-25**	0,83	0,01956	**hsa-miR-17**	0,78	0,04392
**hsa-miR-98**	1,00	0,01966	**hsa-miR-324-5p**	0,73	0,04490
**hsa-miR-106a**	0,71	0,02016	**hsa-miR-320c**	0,82	0,04523
**hsa-let-7a**	1,06	0,02102	**hsa-miR-99a**	0,84	0,04595
**hsa-miR-300**	0,89	0,02126	**hsa-miR-302c**	2,55	0,04598
**hsa-miR-18a**	0,70	0,02210	**hsa-miR-30b**	0,85	0,04622
**hsa-miR-515-3p**	0,89	0,02262	**hsa-miR-28-3p**	0,83	0,04717
**hsa-miR-27b**	0,78	0,02454	**hsa-miR-99**	0,77	0,04962
**hsa-miR-518d-3p**	1,06	0,02537

#### miRNA Expression in ARPE-19 sEVs (sEV miRNA)

As above-mentioned, ARPE-19 cells released a significantly higher number of sEVs after 600 µM H_2_O_2_ exposure than control cells. In contrast, these H_2_O_2_ induced sEVs showed a significant lower miRNA expression compared to control. More concretely, 218 out of 384 sEV miRNAs were detected by the array. However, only 2 out of 218 sEV miRNAs were significantly lower in sEVs released from treated cells (*p* < 0.05) hsa-miR-302a (FC = 1.159) and hsa-miR-122 (FC = 1.576) than in control sEVs (Fig. 5B,D).

### Microarray validation

Among the miRNAs differently expressed in ARPE-19 cells, seven out of 59 presented a significant fold-change. In order to validate this finding, qRT-PCR was performed by using independent RNA samples. We selected three out of seven significant miRNAs (those with the highest differences observed): hsa-miR-205-5p, hsa-miR-521, and hsa-miR-302c; plus a miRNA that was unchanged: hsa-miR-130b, as a control. The outcome confirmed the results obtained, hsa-miR-205-5p, hsa-miR-521 and hsa-miR-302c were down-regulated by 600 μM H_2_O_2_ compared to control (Fig. 5E).

The same procedure, qRT-PCR, was performed using the two sEV miRNAs which expression had changed: hsa-miR-302a and hsa-miR-122. As expected, miRNAs were under-expressed in sEVs released from ARPE-19 cells treated with 600 μM H_2_O_2_ (Fig. 5F).

### Pathway analysis and prediction of miRNA targets regulated by oxidative stress

**Figure 6:**
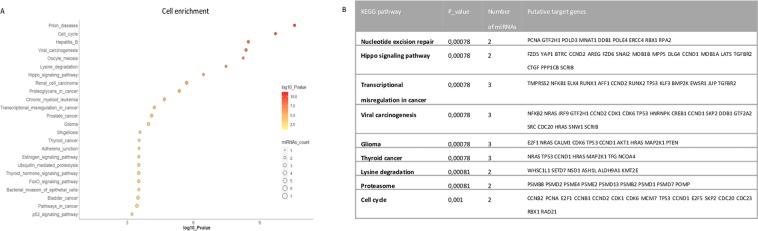
Cell miRNA related pathways after H_2_O_2_ exposure in ARPE-19 cells. Graphic representation of cellular pathways (Y) and log_10_ P-value (X), circle size represents the number of involved miRNAs up to 7 deregulated miRNAs. (**A**) KEGG pathways regulated and putative target genes (**B**).

**Figure 7:**
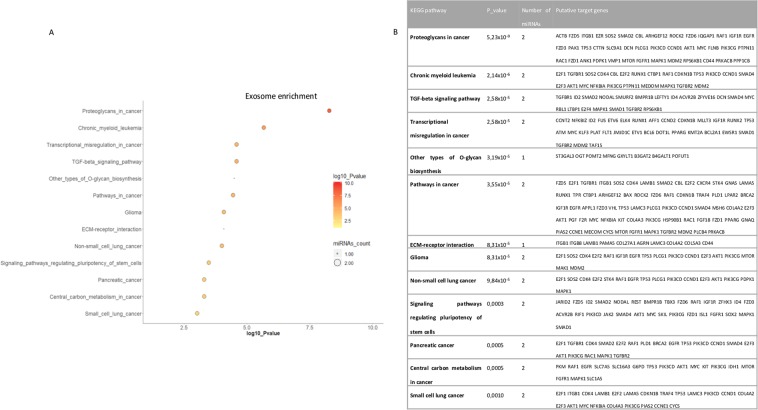
sEVmiRNA related pathways after H_2_O_2_ exposure in ARPE-19 cells (**A**) Graphic representation of cell pathways (Y) and log_10_ P-value (X), circle size represents the number of involved miRNAs up to 2 deregulated miRNAs. (**A**) KEGG pathways regulated and putative target genes (**B**).

Subsequently, the role of the under-expressed miRNAs in treated ARPE-19 cells and in sEVs from treated cells (seven in cells and two in sEVs), was analysed. For that reason, two independent ¨*in silico*¨ analysis were performed in order to determine potential biological processes related to oxidative stress induction. The analysis of the KEGG pathway, regulated by Let-7a, miR-518d-3p, miR-521, miR-338-5p, miR-548b-5p, miR-205, and miR-302c, shows a large number of pathways involving these miRNAs. Among them, cell cycle, adherent junction, p53 signalling pathway, and HIF-1 signalling pathway are the most relevant (Fig. 6A). Both sEV miRNAs, miR-302a and miR-122, are involved in different pathways, such as TGF-beta signalling pathway, FoxO signalling pathway, and cell cycle (Fig. 7A).

### Potential targets of miRNAs

In order to identify biological functions of the validated miRNAs and to select their putative targets, two programs were handled: DIANA TOOLS mirPath and Target Scan Human. The outcome on sEV miRNA and Cell miRNA related pathways are not completely equivalent. Most of the significantly involved KEGG are related to cancer or cell cycle pathways (Figs. 6B and 7B).

## Discussion

The study of circulating miRNAs on AMD has been addressed to find biological markers which might help implementing an early diagnosis for the disease, or to find new therapeutic strategies^18^. Although neovascular processes and oxidative stress are well known characters involved on AMD, the origin of the disease is far from being completely understood. Several studies were focused in identifying neovascularization-related miRNAs or oxidative stress-related ones^23–26^. On this line, the use of RPE cell cultures, as ARPE-19 under oxidative challenges (eg. H_2_O_2_, rotenone or EtOH), results useful to study the RPE response to oxidative stimuli^27–29^.

As mentioned earlier, 600 µM was the highest H_2_O_2_ concentration used which increased ROS levels without generating early apoptosis (800 µM H_2_O_2_ exposure significantly increased early apoptosis). Other authors have used different H_2_O_2_ ranges (200-600 µM) and some differences can be found in terms of cell viability (XTT or MTT), apoptosis and ROS production^7,27,30^. Plausibly, the differences on time exposure (12–24 h) or cell confluence level could explain these discrepancies.

Fitting with previous data, pro-oxidant challenge resulted on significant high sEVs release from ARPE-19 cells. It is well documented that EtOH or high glucose conditions resulted in the same response increasing sEVs release from ARPE-19 cells^28,31^.

Extracellular or circulating miRNAs can be included on sEVs, associated to lipoproteins or proteins^13^. In this respect, we have just focused on miRNAs contained as sEVs cargo (sEV miRNA). In spite of the fact that in sEVs only 218 out of 384 miRNAs could be detected, only two sEV miRNAs were significantly high in control released sEVs than in sEVs from treated cells. The hsa-miR-302a and hsa-miR-122 have already been seen in sEVs confirming our results^32–35^.

When comparing the two sEV miRNAs, hsa-miR-302a and hsa-miR-122, to those miRNAs on ARPE-19 cells (Cell miRNA), no matches could be found. Surprisingly, the sEV miRNA hsa-miR-302a and the Cell miRNA hsa-miR-302c belong to the miR 302/367 *La-related protein 7* (*LARP7*) intragenic cluster. This includes hsa-miR-367, hsa-miR-302d, hsa-miR-302a, hsa-miR-302c, and hsa-miR-302b. This cluster is involved in several processes coordinating proliferation, differentiation, pluripotency maintenance, and cell reprogramming^36^. Moreover, the cluster, regulates TGF pathway, PI3K–AKT and BMP cell signaling^37–39^. Additionally, hsa-miR-302a acts as a tumor suppressor^40^ and repressor of cell division, and more concretely, VEGFA is one of the direct targets for this miRNA^41^. In addition, hsa-miR-122 has been related to VEGFC^42^. Interestingly, low hsa-miR-302a/hsa-miR-122 expression levels are inversely related to VEGF levels in hepatocellular carcinoma, promoting vascular changes^43,44^. In addition, miR-122 seems to have a role against oxidative stress, since the use of pre-miR-122 protects from H_2_O_2-_induced oxidative stress^45^, targeting the mitochondrial ribosomal protein S11^46^.

Recent data from other groups indicate how diverse oxidative insults -EtOH or high glucose, and now H_2_O_2_ - lead to increased sEVs release from ARPE-19 cells^28,31^. Furthermore, those oxidative-induced sEVs were capable of promoting neovascular processes in endothelial cell cultures, whereas control-released sEVs inhibited this phenomenon^28,31^. In view of these findings, we hypothesize that sEVs hsa-miR-122 and hsa-miR-302a decreased levels could play a role on angiogenesis involving oxidative stress related pathways.

Several reports on AMD or experimental AMD, have reported significant changes on miRNAs (see Table 2). Among them, hsa-miR-23a is present in five reports^7,16,17,42,47^. This miRNA seems to be downregulated in both AMD patients and H_2_O_2_-treated ARPE-19 cells. In fact, H_2_O_2_ induced-apoptotic cell death is significantly observed in ARPE-19 cells after hsa-miR-23a inhibition^7^. Fitting with this, we found significant expression differences on hsa-miR-23a after H_2_O_2_ exposure (FC = 0.79). Table 2 summarizes those Cell miRNAs matching with previous reports on AMD or AMD experimental models. Besides, there are reports that locate the sEVS-miRNAs in eye tissue. MiR-302 expression was observed already in RPE cells by Li and collaborators, playing an important role in the RPE differentiation^48^. Other authors observed a miR-122 differential expression on canine retinas^49^, in aqueous humor^50^ and are related in fact with diabetic retinopathy^51^. In agreement with others, up/down regulated miRNAs are related to several and different cell signaling pathways. After seeing the results in both sEV miRNA and Cell miRNAs related pathways, cancer related pathways are commonly involved in many of the deregulated miRNAs. More research must be performed on these generic pathways to determine the concrete role of these cell signaling routes.

**Table 2:** Relevant miRNAs identified on eye disorders.

miRNA	Reference
hsa-miR-139-3p	Szemraj *et al*. and Ertekin *et al*.^17,52^
hsa-miR-192	Grassmann *et al*. and Ertekin *et al*.^24,52,53^
hsa-let-7c	Ren *et al*. and Szemraj *et al*.^17,19^
hsa-miR-18b	Ren *et al*. and Wang *et al*.^19,54^
hsa-miR-183	Karali *et al*., Loscher *et al*. and Xiang *et al*.^55–57^
hsa-miR-23a	Li *et al*., Lin *et al*., Romano *et al*., Szemraj *et al*. and Zhou *et al*.^7,16,17,47,58^
hsa-miR-27a	Ren *et al*.; Romano *et al*.; Szemraj *et al*.^16,17,19^
hsa-miR-25	Ren *et al*. and Zhang *et al*.^19,59^
hsa-miR-106a	Ertekin *et al*.^52^
hsa-let-7a	Ertekin *et al*. and SanGiovanni *et al*.^52,60^
hsa-miR-27b	Ertekin *et al*. and Howell *et al*.^30,52^
hsa-miR-518d-3p	Dinç *et al*.^27^
hsa-miR-92a	Dinç *et al*., Howell *et al*., Desjarlais *et al*. and Walz *et al*.^27,30,61,62^
hsa-miR-361-5p	Grassmann *et al*. and Szemraj *et al*.^17,24^
hsa-miR-335	Ertekin *et al*. and Grassmann *et al*.^24,52^
hsa-miR-30c	Ren *et al*.^19^
hsa-miR-28-5p	Ren *et al*.^19^
hsa-miR-29a	Ertekin *et al*., Howell *et al*., Walz *et al*., Zhang *et al*. and Zhang *et al*. (2)^30,52,62–64^
hsa-miR-205	Ménard *et al*.^4^
hsa-miR-17	Ertekin *et al*., Barber *et al*. and Tian *et al*.^5,18,52^
hsa-miR-324-5p	Ertekin *et al*.^52^
hsa-miR-302c	Howell *et al*.^30^
hsa-miR-30b	Dinç *et al*., Romano *et al*., Mazzeo *et al*. and Haque *et al*.^16,27,65,66^
hsa-miR-28-3p	Howell *et al*.^30^

As a conclusion, H_2_O_2_ significantly increased sEVs release from ARPE-19 cells compared to control cells. Paradoxically, the miRNA sEVs cargo (hsa-miR-302a and hsa-miR-122) resulted in significantly lower in H_2_O_2_-induced sEVs compared to control. Since hsa-miR-302a and hsa-miR-122 regulates vasculogenic targets, these results support those on ARPE-19 cells indicating that oxidative-induced sEVs promote angiogenesis^28,31^.

## Material and Methods

### Cell culture

Arising retinal pigment epithelium (ARPE-19) human cell line was obtained from American Type Culture Collection (ATCC, Barcelona, Spain) at passage 19. ARPE-19 cells were cultured in Dulbecco’s modified Eagle’s DMEM/F12 (Invitrogen, Carlsbad, CA, USA), as previously described^21^. Cells were used until passage 30. Cells were cultured to 80–90% confluence at a starting density of 1 × 10^6^ cells/cm^2^ in different plates depending on the technique. After 2 days, the cells were treated for 24 h with 600 µM H_2_O_2_ (Scharlau, Senmenat, Spain), using filtered media with 1% of Fetal Bovine Serum, exosome-depleted (FBS; Thermo Fisher Scientific, Gibco, USA). Cells and supernatant were collected and preserved for future experiments.

### Determination of intracellular ROS

Intracellular ROS levels were measured using dihydroethidium, (DHE; Thermo Fisher Scientific, Waltham, MA, USA), which is a superoxide indicator. This molecule has a blue fluorescence, but, when oxidized to ethidium, it stains DNA in red. ARPE-19 cells were seeded at 6 × 10^3^ cells/well in a 96 well plate. Cells were rinsed with PBS (phosphate-buffered saline) twice and incubated with 5 μM of DHE during 30 min at 37 °C and 5% CO_2_. ROS levels were measured by a fluorescence multiplate reader (Victor X5; Perkin Elmer) excited at 518 nm and read at 605 nm.

### Apoptosis detection

Number of dead cells by apoptosis or necrosis was measured by flow cytometry using the FITC Annexin V Apoptosis detection Kit (Immunostep, Salamanca, Spain) that can discriminate live cells from those in early apoptosis, late apoptosis, or necrosis. A total of 10,000 cells per condition were analysed using the FACS Verse (Beccton Dikinson, New Jersey, USA). Four populations are detected: unmarked Annexin (−)/Propidium Iodide (−) are live cells; double marked Annexin (+)/Propidium Iodide (+) represent apoptotic cells; simple marked Annexin (+)/Propidium Iodide (−) are early apoptotic cells; and simple marked Annexin (−)/Propidium Iodide (+) are necrotic cells.

### sEVs isolation and size distribution

sEVs isolation was performed by successive ultracentrifugation as previously reported^21^. The sEVs pellet was stored at 4 °C until further processing in PBS solution. For microarray assay, sEVs were isolated using ExoQuick-TC (Systems Biosciences, Mountain View, CA, USA) following the manufacturer’s instructions. sEVs identity was confirmed by the nanoparticle tracking system NanoSight NS300 following manufacturer’s protocols (Malvern Instruments, Malvern, UK).

### Electron microscopy

sEVs pellets were resuspended in PBS and ultracentrifuged at 120.000 × g for 70 min at 4 °C. After that, approximately 10 µg of the samples were resuspended in PBS on parafilm. The sample was fixed by depositing a drop of 2% Paraformaldehyde on the parafilm and placing the grid (Mesh with Formvar) on top of the drop. Negative staining was performed with 2% uranyl acetate. Photomicrographs were obtained using the transmission electron microscope FEI Tecnai G2 Spirit (FEI Europe, Eindhoven, Netherlands) using a digital camera Morada (Olympus Soft Image Solutions GmbH, Münster, Germany). EVs were identified under the microscope solely based on size and morphology.

### RNA isolation and miRNA expression analysis

To perform microarray analysis, ARPE-19 cells from 4 separate cultures were exposed to control and H_2_O_2_ 600 µM treatment for 24 h. Total RNA was extracted using SeraMir Kit (System Biosciences, Mountain View, CA, USA) according to manufacturer’s instructions. Therefore, four microarrays were performed for each condition: control, ARPE-19 cells exposed to H_2_O_2_ 600 µM, sEVs released by control cells and sEVs released by ARPE-19 cells exposed to H_2_O_2_ 600 µM.

Total RNA quantity and quality (260/280 absorbance ratio) were assessed using NanoDrop 2000 (Thermo Fisher Scientific, Waltham, MA, USA). Total RNA was reversely transcribed (cDNA synthesis) using PeqSTAR 96 Universal Gradient (PeqLAb, Erlangen, Germany) under the following conditions: 60 °C/5 min, RT/2 min, 42 °C/30 min, 95 °C/10 min and 15 °C/hold. Real-time quantitative PCR (qPCR) was performed using 384 well SeraMir Profiler using RT-PCR QuantStudio^TM^ 3 y 4 system (Thermo Fisher Scientific, Waltham, MA, USA) with the appropriate temperature cycles 50 °C/2 min, 95 °C/10 min, 40 ciclos; 95 °C/15 s, 6 °C/1 min. The miRNA expression values were calculated using three endogenous controls: RNU43, RNU1Q and RNU6. The expression was calculated according to the 2^−∆∆Ct^ method.

### Array analysis

We analyzed miRNA expression differences between ARPE-19 control cells and ARPE-19 treated with H_2_O_2_. Moreover, differences in miRNA expression between EVs released by ARPE-19 cells treated and EVs released by ARPE-19 control cells were also studied. Differences were analyzed using a t-test study from *genefilter* package from R Bioconductor. *P-values* were adjusted by the Benjamini-Hochberg method. MiRNAs that presented an adjusted p-value < 0.05 were considered statistically significant. Significantly modified miRNAs from different samples were represented in a hierarchical clustering heatmap representation. Heatmaps were performed using *heatmap*.*3* package from R Bioconductor.

### Analysis of miRNA target genes

*In silico* analysis of the pathways in which the miRNAs regulated by H_2_O_2_ were involved using DIANA TOOLS mirPath v.3 algorithm (http://snf-515788.vm.okeanos.grnet.gr/). Moreover, we carry out an analyse of the putative miRNAs target using TargetScanHuman (http://www.targetscan.org/vert_72/).

### Quantitative real-time PCR validation

Quantitative real time PCR (qRT-PCR) was used to validate the miRNA expression profile of the selected miRNAs in an independent sample set. The RNA was isolated from ARPE-19 cells by miRNeasy Mini Kit (Qiagen, Hilden, Germany) according to the manufacturer’s instructions. 100–300 ng of RNA were retro transcribed using TaqMan MicroRNA Reverse Transcription Kit (Thermo Fisher Scientific, Waltham, MA, USA) using specific TaqMan RT primers and the thermocycler PeqSTAR 96 Universal Gradient (PeqLAB, Erlangen, Germany), the cycles used were 16 °C/30 min, 42 °C/30 min, 85 °C/5 min and 4 °C/infinity. Quantitative real time PCR was performed using TaqMan™ microRNA Assays (Thermo Fisher Scientific, Waltham, MA, USA) with TaqMan Gene Expression master Mix (Thermo Fisher Scientific, Waltham, MA, USA) and RT-PCR Roche 234 LighterCycler 480 with the appropriate temperature cycles (50 °C/2 min, 95 °C/10 min, 40 cycles: 95 °C/15 s and 60 °C/1 min). Normalisation was performed with RNU6B snoRNA and RNU43 snoRNA. Relative expression was calculated as 2^−ΔΔCt^.

### Statistical analysis

The results of each experiment are presented as mean ± SEM. Statistical significance was determined using t-test and 2-way-ANOVA.

## Data Availability

The datasets generated during and/or analysed during the current study are available from the corresponding author on reasonable request.
